# Which NK cell populations mark the high burden of CMV present in all HIV patients beginning ART in Indonesia?

**DOI:** 10.1186/s12981-022-00439-2

**Published:** 2022-03-15

**Authors:** Ibnu A. Ariyanto, Riwanti Estiasari, Birry Karim, Ika Praseya Wijaya, Budiman Bela, Amin Soebandrio, Patricia Price, Silvia Lee

**Affiliations:** 1 Doctoral Program of Biomedical Science, Faculty of Medicine, Universitas Indonesia, Jakarta, Indonesia; 2 Virology and Cancer Pathobiology Research Center, Faculty of Medicine, Universitas Indonesia, Jakarta, Indonesia; 3 Department of Neurology, Faculty of Medicine, Universitas Indonesia, Jakarta, Indonesia; 4 Cardiology Division, Internal Medicine, Universitas Indonesia and Cipto Mangunkusomo Hospital, Jakarta, Indonesia; 5 Eijkman Institute for Molecular Biology, Jakarta, Indonesia; 6 Curtin Medical School and the Curtin Health Innovation Research Institute (CHIRI), Curtin University, Bentley, Perth, 6102 Australia; 7 Department of Microbiology, Pathwest Laboratory Medicine, Perth, Australia

**Keywords:** CMV, NK cells, HIV, ART

## Abstract

**Background:**

Cytomegalovirus (CMV) has been linked with cardiovascular disease (CVD) in populations where some individuals are seronegative. However, effects of CMV are unclear in HIV patients who all have high levels of CMV antibodies. Other metrics of their CMV burden are needed. Amongst transplant recipients, CMV drives the expansion of NK cell populations expressing NKG2C and/or LIR1 and lacking FcRγ.

**Methods:**

Indonesian HIV patients (n = 40) were tested before ART and after 6 months, with healthy local controls (n = 20). All patients had high CMV antibody titres. 52% started therapy with CMV DNA detectable by qPCR, providing a crude measure of CMV burden. Proportions of CD56^Hi^ or CD56^Lo^ NK cells expressing FcRγ, NKG2C or LIR1 were determined flow cytometrically. CVD was predicted using carotid intimal media thickness (cIMT). Values were correlated with levels of CMV antibodies on ART.

**Results:**

Patients had low proportions of CD56^Lo^ and more CD56^Hi^ NK cells. However proportions of FcRγ^−^ NK cells were *lowest* in patients with CMV DNA, and cIMT values related *inversely* with FcRγ^−^ NK cells in these patients. Percentages of NKG2C^+^CD56^Lo^ NK cells were similar in patients and controls, but rose in patients with CMV DNA. Proportions of NKG2C^+^ CD56^Hi^ NK cells correlated with levels of CMV antibodies in CMV DNA-negative patients.

**Conclusions:**

We show that the very high burdens of CMV in this population confound systems developed to study effects of CMV in other populations. FcRγ^−^ NK cells may be depleted by very high CMV burdens, but NKG2C and antibody levels may be informative in patients on ART.

**Supplementary Information:**

The online version contains supplementary material available at 10.1186/s12981-022-00439-2.

## Introduction

Natural killer (NK) cells are implicated in the control of cytomegalovirus (CMV) infections. Moreover, CMV can shape the NK cell repertoire driving the expansion of specific NK subpopulations [1]. The balance reached in this feedback loop will depend upon other forces that shape the immune system of the host with impact upon NK cell populations.

NK cells with the phenotype CD56^Lo^CD16^Hi^ comprise 90% of the total NK cell population. These cells are mature and cytotoxic. CD56^Hi^CD16^Lo^ NK cells comprises the residual 10% of the population and are less mature, less cytotoxic and exhibit more potent cytokine release upon stimulation [2]. NK cell function is regulated by activating and inhibitory receptors [3]. Inhibitory receptors, such as LIR1, interact with MHC class 1 molecules, preventing attacks on ‘’self’’ cells. Consequently, a lack of MHC class 1 expression leads to NK cell activation via activating receptors, including NKG2C [4]. FcRγ is an immunoreceptor tyrosine-based activation motif-containing adaptor protein responsible for transducing signals through activating NK cell receptors such as CD16 (FcγRIIIa) and acting as a chaperone for these receptors [5]. We and others have described increased proportions of NK cells lacking FcRγ and expressing NKG2C and/or LIR1 in CMV-seropositive transplant recipients [1, 6]. However, effects of CMV are less clear in HIV patients.

NK cells from Australian HIV patients stable on long-term antiretroviral therapy (ART) responded poorly to in vitro stimulation, but this could not be attributed to CMV as responses were low in CMV-seronegative healthy controls. Moreover, HIV (and not CMV) increased the expression of CD57 on CD56^Lo^ NK cells [7]. In the same patient population, proportions of CD56^Hi^ NK cells correlated inversely with current CD4 T-cell counts, and perforin expression in CD56^Hi^ NK cells was higher in HIV patients than controls. Hence, increased proportions and cytolytic function of CD56^Hi^ NK cells may partially compensate for CD4 T-cell deficiency [8]. FcRγ was not assessed in these studies but was subsequently examined in Australian patients beginning ART. Proportions of FcRγ^−^ NK cells were not associated with NK cell, T-cell or monocyte activation, so different factors may drive CD56^Lo^ FcRγ^−^ NK cell expansion and immune activation in HIV^+^ individuals. Patients retained elevated levels of CMV-reactive antibodies on ART, but these did not predict proportions of CD56^Lo^ FcRγ^−^ NK cells [9].

Epidemiological studies have associated persistent CMV infection with age-related diseases, such as cardiovascular disease (CVD) in individuals with no history of acute (end organ) CMV disease. Atherosclerosis is a common cause of CVD and is characterised by the accumulation of lipids and cholesterol, creating plaques in the arterial walls [10]. The resultant narrowing of the arteries can lead to coronary heart disease and stroke. CMV DNA has been reported in 82% of atherosclerotic plaques, with positive correlations between CMV viral load and proportions of effector memory T-cells in the plaques [11]. Proportions of LIR1^+^ and/or FcRγ^−^ NK cells induced by CMV correlated inversely with flow-mediated dilatation (i.e., vascular endothelial function) in renal transplant recipients and healthy adults [12]. Of seven recipients with detectable CMV DNA in plasma, we observed the highest frequency of NK cells expressing NKG2C and LIR1 without FcRγ in the individual with the highest burden of CMV [13]. Thus, NK cell profiles may be used as a metric of the burden of CMV as it impacts upon CVD in this setting.

Here, we assess how CMV and HIV change NK cell profiles in patients starting ART with a very high burden of CMV, and how this may impact upon an early marker of cardiovascular health—carotid intimal media thickness (cIMT). This was achieved in the JakCCANDO cohort recruited in Jakarta, Indonesia, and followed during their first year on ART. All patients were CMV-seropositive with very high antibody titres, and 50% had CMV DNA detectable with a simple in-house qPCR when they began ART. Factors impacting upon cIMT have been described previously [14]. Here we compared adaptive NK cell phenotypes in CMV DNA+ and CMV DNA− patients and considered inflammatory biomarkers invoked by viral infections [C-reactive protein (CRP) and soluble interferon receptor (sIFNR)-α/β]. The latter can regulate the biological activity of IFNα/β through competition at high concentrations and stabilisation at lower concentrations [15]. Levels of sIFNRα/β may be downregulated in HIV patients with poor control of HIV on ART—potentially increasing the antiviral activity of IFNα/β [16]. Associations with CMV and NK cell activation have not been considered in this context.

## Materials and methods

### Study subjects

The JakCCANDO study (Jakarta CMV Cardiovascular ART Neurology Dentistry Ophthalmology) examined 82 HIV patients starting ART at the HIV/AIDS clinic of Cipto Mangunkusumo Hospital (Jakarta, Indonesia). The trial was retrospectively registered. Here, we describe 40 patients from the cohort before ART (V0) and after 6 months (V6). All had < 200 CD4 T-cells/μl so their HIV infections are probably long-standing. Patients received zidovidine/lamivudine/nevirapine (40%), zidovudine/lamivudine/efavirens (25%), nevirapine/lamivudine/tenofovir (12.5%), nevirapine/stavudin/lamivudine (5%) or stavudine/lamivudine/efavirens (2.5%). Control donors (n = 20), matched group-wise with the patients by gender, age and ethnicity, were sampled once. The Faculty of Medicine (Universitas Indonesia) and Cipto Mangunkusumo Hospital ethics committees approved the study (31/H2.F1/ETIK/2012 and 26/H2.F1/ETIK/2013). Written informed consent was obtained from all participants. CD4 T-cell counts were determined by routine flow cytometry, and plasma HIV RNA was evaluated using a Cobas Amplicor Monitor (Roche Molecular Diagnostics, Pleasanton, CA). Peripheral blood mononuclear cells (PBMC) were isolated from whole blood by Ficoll density centrifugation and cryopreserved in liquid nitrogen. Plasma and PBMC-depleted buffy coats were stored at − 80 °C. Carotid Doppler sonography was used to evaluate arterial circulation using B-mode, colour flow and velocity measurements. The outcome is expressed as carotid intimal medial thickness (cIMT) assessed when the artery was in the diastolic phase [14].

### Quantitation of CMV-reactive antibody, CMV DNA and inflammatory biomarkers

CMV-reactive antibody was quantified using 96-well plates coated with a lysate of human foreskin fibroblasts infected with CMV strain AD169, or recombinant CMV Immediate Early 1 (IE-1) protein (Miltenyi Biotech; Cologne, Germany). Plates were coated overnight at 4 °C, blocked with 5% bovine serum albumin, and plasma samples were added (pre-diluted 1:10,000 for CMV lysate and 1:300 for CMV IE-1, followed by three-fold dilutions). Bound IgG was detected using goat anti-human IgG-horseradish peroxidase, followed by tetramethylbenzidine substrate (Sigma-Aldrich; St Louis, MI). Levels of CMV-reactive antibodies were determined relative to a standard plasma pool assigned a value of 1000 arbitrary units (AU). The protocol provides accurate quantitation in the high range [17]. CMV DNA was detected in buffy coats by qPCR using primer and probe sequences targeting the UL54 gene as described previously [14]. CRP and sIFNRα/β levels in plasma were quantitated using commercial ELISA reagents [17].

### Natural killer cell immunophenotyping

Cryopreserved PBMC were thawed, washed twice with phosphate buffer saline and stained with Fixable Viability Stain 620 (1:2500) for 15 min at room temperature. This was followed by antibodies detecting surface markers (15 min, 4 °C)—anti-CD3 BV500 (clone UCHT1), anti-CD56 PE-Cy7 (clone B159), anti-CD57 BV421 (clone NK-1), anti-CD16 APC-H7 (clone 3G8), anti-CD57 APC (clone NK-1) (BD Bioscience; San Jose, CA), anti-NKG2C APC (clone 134591) (R&D Systems; Minneapolis, MN), anti-LIR-1 PE (clone HP-F1) (eBioscience; San Diego, CA), and anti-FcεRIγ FITC (γ subunit) (Millipore; Temecula, CA). Cells were then fixed with BD Cytofix/cytoperm (BD Bioscience) and washed before acquisition on an 8-colour FACS Canto II cytometer. Data was exported from BD FACS Diva software into FCS3.0 files and analysed using FlowJo V10.6.2 (BD Bioscience). FCS data were cleaned using FlowAI plugins to exclude electronic noise [18], and doublets and dead cells were excluded [19]. Mean fluorescent intensity (MFI) was used when it was difficult to gate distinct populations in all samples. Gating strategies are summarised in Additional file 1: Fig. S1.

### Statistical analyses

As most parameters deviated from normal distributions (Shapiro–Wilk test), all data were analysed using non-parametric statistics and presented as median (range). Mann–Whitney unpaired tests were used to compare groups, and Wilcoxon paired tests were used to assess changes over time. Spearman’s Rank Tests were used to analyse correlations. Statistical analyses were performed using GraphPad Prism 8 (GraphPad, San Diego, CA) and p ≤ 0.05 was accepted as a significant difference.

## Results

### Inflammation and a high burden of CMV persisted on ART

Total CD4 T-cell counts rose, and HIV viral loads declined by V6, but levels of the inflammatory markers CRP and sIFNRα/β were maintained (Table 1, Panel 1). Levels of antibody reactive with CMV lysate increased on ART and were markedly higher than in healthy controls. These findings match those reported with the original JakCCANDO cohort (n = 82) [14, 17]. As 52% of the original JakCCANDO cohort were CMV DNA+ at V0, we selected 20 HIV patients with and 20 without CMV DNA for this study. CMV DNA+ and CMV DNA− patients had similar levels of antibodies reactive with CMV lysate at V0 [median (range): 4.1 (3.4–5.3) *vs* 4.1 (3.2–4.6); p = 0.27] and only slightly higher levels at V6 [4.5 (3.5–5.2) *vs* 4.26 (3.1–4.8); p = 0.054]. Levels of CMV antibody were similar in patients with HIV RNA levels below or above 500 copies/ml at V6 (data not shown), so the high burden of CMV could not be linked with poor adherence to therapy. cIMT values were within the normal range in most patients and did not change significantly on ART.

**Table 1:** JakCCANDO HIV patients retain high levels of CMV-reactive antibodies on ART, but cIMT values remain in the normal range

	Healthy controls	HIV V0	HIV V6	p value		
	n = 20	n = 40	n = 40			
	A	B	C	A *vs* B	A *vs* C	B *vs* C
Clinical and demographic features						
Age (years)	32 (18–45)	32 (19–47)	–	0.43^a^	–	–
Sex (M/F)	12/8	30/10	–	0.25^b^	–	–
HIV RNA (Log_10_ copies/µL)	–	5.0 (2.9–6.4)	0.76 (0–5.0)	–	–	**0.0001**^**c**^
CD4 T-cells (cells/µL)	–	68 (2–199)	225 (6–516)	–	–	**0.0001**
CMV Lysate antibody (Log_10_ AU)	3.3 (2.8–4.8)	4.1 (3.2–5.2)	4.3 (3.1–5.2)	**0.02**^**a**^	**0.003**^a^	**0.03**
CMV IE-1 antibody (Log_10_ AU)	2.3 (1.1–3.8)	2.7 (1.7–3.8)	2.7 (1.2–3.8)	0.10	0.16	0.25
CRP (µg/mL)	–	1.9 (0.007–50)	2.2 (0.004–54)	–	–	0.97
sIFNRα/β (ng/mL)	–	4.4 (2.5–8.7)	4.4 (1.8–6.8)	–	–	0.67
Right cIMT (mm)	< 0.70^d^	0.58 (0.39–0.77)	0.57 (0.39–0.77)^e^	–	–	0.08
Left cIMT (mm)	< 0.70^d^	0.57 (0.45–0.83)	0.51 (0.32–0.70)	–	–	0.24
NK cell phenotypes						
CD16^+^ (% of CD56^Hi^)	58 (23–67)	58 (28–75)	62 (11–81)	0.15	0.06	0.48
CD16^+^ (% of CD56^Lo^)	81 (42–96)	81 (37–95)	80 (48–96)	0.48	0.60	0.24
LIR1^+^ (% of CD56^Hi^)	0.6 (0–1.6)	0.4 (0–4.6)	0.5 (0–10)	0.48	0.59	**0.01**
LIR1^+^ (% of CD56^Lo^)	1.6 (0.3–5.9)	2.2 (0.06–13)	2.4 (0.2–9.4)	0.19	0.09	0.80
CD57^+^ (% of CD56^Hi^)	7.0 (0.7–29)	4.3 (0.15–34)	4.0 (0.1–22)	0.26	0.10	0.46
CD57^+^ (% of CD56^Lo^)	75 (27–90)	58 (29–89)	60 (18–89)	**0.02**	**0.02**	0.23

Data are presented as median (range). Bold: p value ≤ 0.05

^a^Mann-Whitney tests

^b^Fisher’s exact tests

^c^Paired Wilcoxon tests

^d^As used in clinical care, ^e^ n = 26

### HIV patients had fewer CD56^Lo^ NK cells with no bias towards NK cell subpopulations previously linked with CMV

**Fig. 1:**
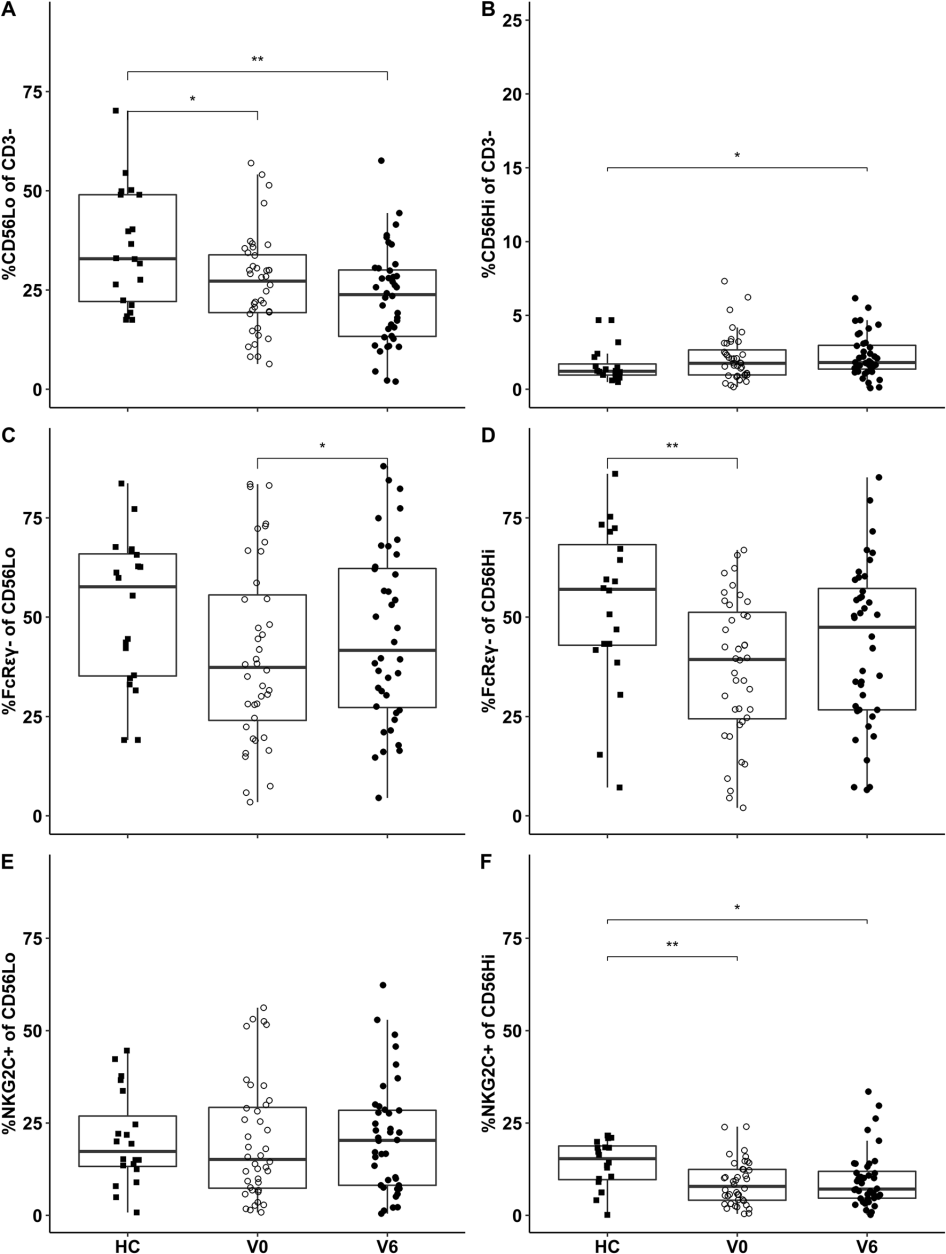
HIV and ART affect NK cell phenotypes. Proportions of CD56^Lo^ NK cells were reduced in HIV patients (**A**), with a small increase in CD56^Hi^ NK cells by V6 (**B**). Proportions of FcRγ^−^ NK cells were low at V0 with a trend towards recovery on ART (**C**, **D**). Proportions of NKG2C^+^ CD56^Lo^ cells were unchanged by HIV and ART (**E**), but HIV reduced expression of NKG2C on CD56^Hi^ NK cells (**F**). Mann–Whitney tests were used to compare patients with healthy controls, and Wilcoxon tests to compare patients at V0 and V6. Vertical lines depict the 95% confidence interval of the median, boxes represent the interquartile range and horizontal lines mark the median. *p < 0.05 and **p < 0.01

Patients displayed reduced proportions of CD56^Lo^ and marginally more CD56^Hi^ NK cells at V0, with no recovery on ART (Fig. 1A, B). We then considered NK cell subpopulations known to be enriched by CMV in transplant recipients and healthy donors [6, 12]. Proportions of NK cells without FcRγ (i.e., FcRγ^−^) were generally low at V0 (Fig. 1C, D). The proportion of CD56^Lo^ NK cells expressing NKG2C was similar in patients and healthy controls, but expression on CD56^Hi^ NK cells was lower in patients (Fig. 1E, F).

Three other NK cell phenotypes were examined (Table 1; Panel 2). Proportions of CD16^+^ NK cells were not significantly affected by HIV or ART. Percentages of LIR1^+^ CD56^Hi^ NK cells rose on ART but were not affected by HIV per se. HIV patients had lower proportions of CD57^+^ CD56^Lo^ NK cells than healthy controls.

### Proportions of CD56^Lo^ NK cells with the FcRγ^−^, NKG2C^+^, and CD57^+^ phenotypes increased on ART in CMV DNA+ patients

**Fig. 2:**
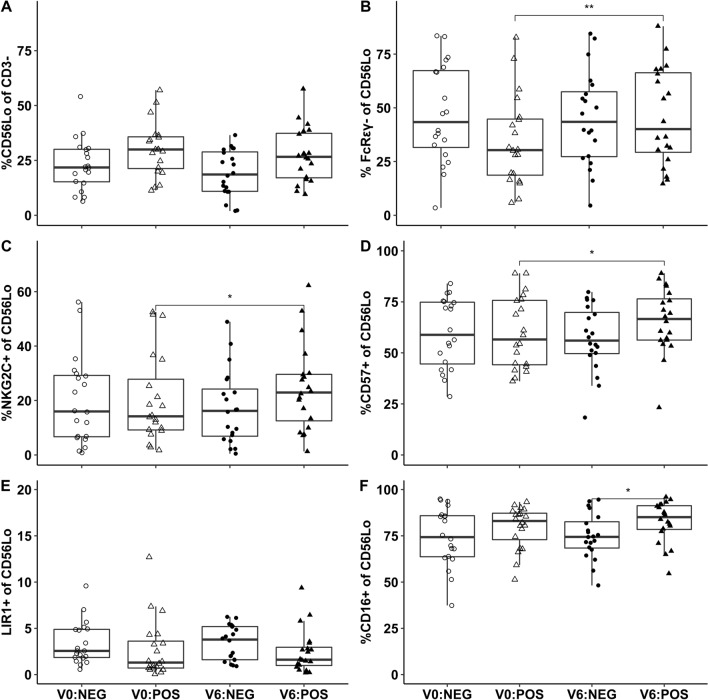
CMV DNA positivity affects subpopulations of CD56^Lo^ NK cells. Proportions of CD56^Lo^ NK cells trended higher in CMV DNA+ group (**A**). FcRγ^−^ (FcRεγ^−^) CD56^Lo^ NK cells were lowest in CMV DNA+ patients at V0 and improved after 6 months of ART (**B**). Proportions of NKG2C^+^ and CD57^+^ NK cells increased on ART in CMV DNA+ patients (**C**, **D**). Proportions of LIR1^+^ NK cells were similar in patients with and without CMV DNA (**E**). Proportions of CD16^+^ CD56^Lo^ NK cells at V6 were higher in CMV DNA+ patients (**F**). Mann–Whitney tests were used to compare patients with and without detectable CMV DNA at V0. Wilcoxon tests were used to compare patients at V0 and V6. Vertical lines depict 95% confidence interval of the median, boxes represent the interquartile range and horizontal lines mark the median. *p < 0.05 and **p < 0.01

We first sought associations between CMV and low proportions of CD56^Lo^ NK cells described in Fig. 1A, and found no significant difference between patients with and without detectable CMV DNA at V0 (Fig. 2A). Proportions of FcRγ^−^ NK cells were lowest at V0 in patients with detectable CMV DNA and improved after six months on ART in these patients (Fig. 2B). Proportions of NKG2C^+^ and CD57^+^ NK cells also increased on ART in CMV DNA+ patients (Fig. 2C, D). Proportions of LIR1^+^ NK cells were similar in patients with and without CMV DNA (Fig. 2E), whilst the proportions of CD16^+^ CD56^Lo^ NK cells at V6 were slightly higher in CMV DNA+ patients (Fig. 2F).

### NK cell populations correlate inversely with levels of CMV-reactive antibodies and sIFNRα/β in patients without detectable CMV DNA at V0

We then addressed whether NK cell subpopulations correlated with plasma levels of antibodies reactive with CMV lysate, or sIFNRα/β (Table 2). Stratification of the cohort by CMV DNA at V0 generated markedly different patterns of association.

**Table 2:** NK cell phenotypes linked with CMV in transplant recipients (FcRγ^−^ and LIR1^+^) do not correlate directly with CMV antibodies or plasma sIFNR α/β in JakCCANDO HIV patients

	CMV DNA+		CMV DNA−	
	V0 *r values*	V6 *r values*	V0 *r values*	V6 *r values*
CMV lysate antibody (log_10_ AU) versus…				
CD56^Hi^ CD3^−^ (% lymphocytes)	− 0.07	− 0.04	− 0.13	0.26
CD57^+^ (%CD56^Hi^)	− 0.29	− 0.31	− 0.07	− 0.05
FcRγ^−^ (%CD56^Hi^)	0.02	− 0.24	0.04	− 0.37
LIR1^+^ (%CD56^Hi^)	0.13	− 0.3	− 0.4	− 0.42
NKG2C^+^ (%CD56^Hi^)	− 0.44	− 0.21	**0.60**	0.27
CD56^Lo^ CD3^−^(% lymphocytes)	− 0.39	0.002	**− 0.64**	− 0.14
CD57^+^ (%CD56^Lo^)	0.05	0.19	− 0.14	− 0.45
FcRγ^−^ (%CD56^Lo^)	− 0.26	− 0.11	− 0.13	**− 0.57**
LIR1^+^ (%CD56^Lo^)	0.06	− 0.29	0.08	0.19
NKG2C^+^ (%CD56^Lo^)	− 0.37	− 0.1	0.39	0.11
Plasma sIFNRα/β versus…				
CD56^Hi^ CD3^−^(% lymphocytes)	− 0.13	− 0.33	0.43	0.44
CD57^+^ (%CD56^Hi^)	− 0.05	0.04	**− 0.45**	0.21
FcRγ^−^ (%CD56^Hi^)	0.002	− 0.26	**− 0.56**	**− 0.47**
LIR1^+^ (%CD56^Hi^)	− 0.25	0.03	− 0.26	− 0.34
NKG2C^+^ (%CD56^Hi^)	0.05	− 0.11	− 0.16	0.05
CD56^Lo^ CD3^−^(% lymphocytes)	− 0.01	0.1	− 0.22	0.21
CD57^+^ (%CD56^Lo^)	− 0.09	**0.45**	− 0.43	− 0.26
FcRγ^−^ (%CD56^Lo^)	− 0.05	0.27	**− 0.57**	− 0.4
LIR1^+^ (%CD56^Lo^)	− 0.09	− 0.27	0.35	0.22
NKG2C^+^ (%CD56^Lo^)	− 0.16	0.25	− 0.23	0.09

Spearman rank correlation tests were used to compare plasma biomarkers with NK cell profiles, Bold: p value ≤ 0.05

Levels of sIFNRα/β correlated with CMV antibody in CMV DNA− patients at V6 (r = 0.54, p = 0.02), but not at V0 (r = 0.16, p = 0.51). However, we noted a correlation between sIFNRα/β and CMV IE-1 antibody levels in CMV DNA+ patients at V0 (r = 0.47, p = 0.04) (data not shown). This would be consistent with a link between sIFNRα/β and current CMV replication. Moreover HIV RNA levels correlated with sIFNRα/β in CMV DNA+ patients at V0 (r = 0.51, p = 0.02) with no correlation in CMV DNA− patients (r = 0.004, p = 0.98, data not shown). Hence CMV and HIV may induce sIFNRα/β, but the finding varies over time on ART and with the burden of CMV.

Proportions of CD56^Lo^ NK cells correlated inversely with CMV lysate antibodies in CMV DNA− patients at V0 (r = − 0.64, p = 0.003), linking with their depletion. There were no significant correlations between CMV-reactive antibodies and CD57^+^, LIR1^+^ or FcRγ^−^ NK cells, except for an inverse association with the latter at V6 in CMV DNA− patients (r = − 0.57, p = 0.01). These populations displayed several inverse correlations with levels of sIFNRα/β in CMV DNA− patients, providing no evidence for their *expansion* by CMV.

In CMV DNA− patients, proportions of NKG2C^+^ NK cells correlated directly with CMV-reactive antibody levels (CD56^Hi^; r = 0.60, p = 0.01; CD56^lo^, r = 0.39, p = 0.10) at V0. As this is consistent with the expansion of NKG2C^+^ NK cells by CMV before ART, it is pertinent that CMV DNA positivity aligned very weakly with heterozygous carriage of a deletion in the gene encoding NKG2C (χ^2^ = 2.5, p = 0.11) [6, 17]. The role of NKG2C in the control of CMV in this context warrants further study.

### Antibody levels suggest an effect of CMV on cIMT after 6 months on ART, whilst NK cell markers fail under high burdens of CMV

**Fig. 3:**
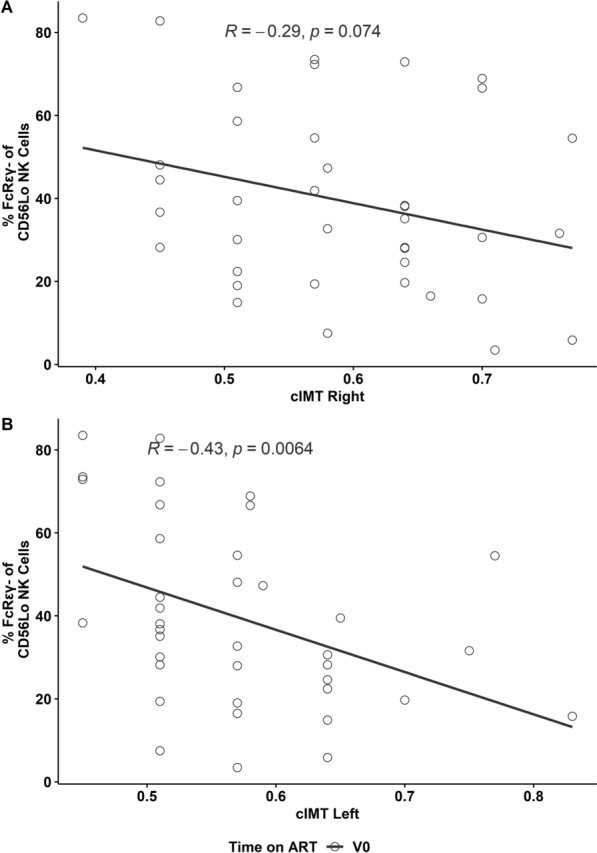
FcRγ^−^ CD56^Lo^ NK cells associate with healthier cIMT values in HIV patients before ART. Proportions of FcRγ^−^ CD56^Lo^ NK cells at V0 associated inversely with right (**A**) and left (**B**) cIMT. Spearmans correlations are shown

Levels of CMV antibody were not correlated with cIMT at V0 (p > 0.17). However antibody levels rise on ART, so CD56^lo^ NK cells lacking FcRγ and/or expressing NKG2C or LIR1 may provide a better metric of the burden of CMV at V0. However at V0, associations between the FcRγ^−^ population and cIMT were *negative* (Fig. 3), so the population may be protective or its induction by CMV may be reversed at high CMV burdens. The latter explanation is favoured because the negative association was confined to individuals whose detectable CMV DNA suggests very high CMV burdens (right: r = − 0.43, p = 0.07; left: r = − 0.50, p = 0.03). At V0, there were no significant associations between cIMT and NK cell expression of NKG2C (p > 0.79) or LIR1 (p > 0.35).

As several patients did not present for cIMT measurements at V6, we have not stratified these data by the detection of CMV DNA. The only associations with cIMT approaching significance were levels of antibodies reactive with CMV lysate (right: r = 0.33, p = 0.11; left: r = 0.36, p = 0.08) and CMV IE-1 (right: r = 0.53, p = 0.01; left: r = 0.44, p = 0.04). Hence antibody levels link a greater CMV burden with poor vascular health after 6 months on ART.

## Discussion

All HIV patients in the JakCCANDO cohort had a high burden of CMV. This is evidenced by the very high levels of CMV-reactive antibody seen in all participants and the detection of CMV DNA by a simple qPCR in 52% of patients [14, 17]. The time of CMV infection was not documented, but was probably in childhood (pre-dating HIV infection). It is also likely that patients underwent reactivations of pre-existing CMV infections prior to ART as they achieved CD4 T-cell counts below 200 cells/μl. Moreover levels of antibody were similar at V0 and V6 in patients with and without detectable CMV DNA [22], so we assume that this grouping divides patients with a moderately high burden of CMV from those with an extremely high burden. In this respect the cohort is distinct from transplant recipients.

Our previous study of NK cell phenotypes in a smaller subset of the JakCCANDO cohort demonstrated low proportions of CD56^+^ NK cells [17]. The first goal of this study was a more precise definition of the NK cell phenotypes generated in 40 JakCCANDO patients before ART and after 6 months. We show that the depletion of CD56^+^ NK cells reflects a loss of CD56^Lo^ cells, with proportions of CD56^Hi^ cells rising slightly.

Levels of the inflammatory markers, CRP and sIFNRα/β, did not decline after six months of ART despite suppression of HIV replication. Whilst type 1 interferons may promote NK cell responses to CMV [20], plasma sIFNRα/β has not been assessed in this context. We found direct associations between levels of sIFNRα/β and levels of HIV RNA in CMV DNA+ patients at V0 and CMV lysate antibody in CMV DNA− patients at V6. This suggests that both viruses can invoke sIFNRα/β.

NK cell responses to CMV in HIV patients have been linked with an accumulation of NK cells expressing CD57 and/or NKG2C [7, 9]. Expression of NKG2C may impact the persistence of CMV as NKG2C^+^ NK cells can present CMV antigen to CD4 T-cells in an HLA-DR dependent pathway [21]. Previous studies link CMV seropositivity per se with the accumulation of NKG2C^+^ NK cells in transplant recipients and healthy adults [6]. Here proportions of NKG2C^+^ NK cells correlated with CMV-reactive antibody levels in CMV DNA− patients at V0. This suggests that quantifiable induction of NKG2C by CMV is a low zone phenomenon that is less apparent when *all* patients and controls are CMV seropositive with high levels of antibodies. Here proportions of CD56^Lo^ NK cells expressing NKG2C were similar in patients and controls, and in patients who were CMV DNA+ or CMV DNA− at V0. This may explain the absence of significant associations between proportions of NKG2C^+^ NK cells and cIMT.

Expression of LIR1 has also been linked with CMV in transplant recipients [6, 12], but LIR1^+^ NK cell populations showed no significant effects of HIV disease or CMV DNA positivity at V0, and proportions did not correlate with CMV antibody levels, sIFNRα/β or cIMT.

An accumulation of CD56^Lo^ FcRγ^−^ NK cells has been described in Australian HIV patients over the first two years of ART, but was not associated with CMV antibody levels or other immune activation markers even though these were markedly elevated [9]. Our longitudinal data reveals low proportions of FcRγ^−^ NK cells at V0, increasing over six months. The increase was most evident in patients who were CMV DNA+ at V0, so it may be driven by CMV once patients are on ART (and not before ART). However, proportions of FcRγ^−^ NK cells were *inversely* related to levels of CMV antibodies and sIFNRα/β in CMV DNA− patients at V6. We also identified negative associations between the FcRγ^−^ population and cIMT at V0, so the NK cell populations may be protective or the induction of these populations by CMV may be reversed at high CMV burdens. Together these findings invalidate CD56^Lo^ FcRγ^−^ NK cells as a metric of CMV in HIV patients with a high burden of CMV, and emphasise the more substantial influence of HIV on the population.

A limitation of our study is that CMV DNA status was only assessed before ART. However, a follow-up of the available buffy coats revealed CMV DNA in 13/23 patients at V3 and 1/3 patients at V12. CMV does not replicate in granulocytes, so its presence in buffy coats may reflect debris from CMV infection sites taken up by granulocytes. We also acknowledge that CMV-reactive antibody is an imperfect metric of the viral burden as levels rise on ART. However after 6 months on ART, levels of antibodies reactive with CMV lysate and CMV IE-1 correlated with cIMT (an early marker of CVD), so antibody levels link a greater CMV burden with poor vascular health on ART.

## Conclusions

We have described lowered proportions of CD56^Lo^ NK cells in Indonesian HIV patients and show that they do not improve on ART. The detection of CMV DNA at V0 did not differentiate the NK cell profiles. This may reflect the high CMV burden in all HIV patients. We show that FcRγ^−^ NK cells may be depleted by very high CMV burdens, but NKG2C and antibody levels may be informative. Hence phenotypes identified as “footprints” of CMV on NK cells from transplant recipients performed poorly in this respect in HIV patients.

## Supplementary Information

**Additional file 1.** Gating strategies used to 1) exclude electronic noise; 2) define live and single cells; and 3) define NK cell sub-populations using antibodies defined in Materials and Methods.

## Data Availability

Data and materials are available upon request.

## References

[1] Barnes S, Schilizzi O, Audsley KM, Newnes HV, Foley B (2020). Deciphering the immunological phenomenon of adaptive natural killer (NK) cells and cytomegalovirus (CMV). Int J Mol Sci.

[2] Knox JJ, Cosma GL, Betts MR, McLane LM (2014). Characterisation of T-bet and eomes in peripheral human immune cells. Front Immunol.

[3] Pegram HJ, Andrews DM, Smyth MJ, Darcy PK, Kershaw MH (2011). Activating and inhibitory receptors of natural killer cells. Immunol Cell Biol.

[4] Mistry AR, O’Callaghan CA (2007). Regulation of ligands for the activating receptor NKG2D. Immunology.

[5] Seidel UJE, Schlegel P, Lang P (2013). Natural killer cell mediated antibody-dependent cellular cytotoxicity in tumor immunotherapy with therapeutic antibodies. Front Immunol.

[6] Makwana NB, Foley B, Lee S, Fernandez S, Irish AB, Price P (2016). Asymptomatic CMV infections in long-term renal transplant recipients are associated with the loss of FcRγ from LIR-1^+^ NK cells. Eur J Immunol.

[7] Affandi JS, Montgomery J, Lee S, Price P (2015). HIV patients stable on ART retain evidence of a high CMV load but changes to natural killer cell phenotypes reflect both HIV and CMV. AIDS Res Ther.

[8] Tan DBA, Fernandez S, French M, Price P (2009). Could natural killer cells compensate for impaired CD4+ T-cell responses to CMV in HIV patients responding to antiretroviral therapy?. Clin Immunol.

[9] Hearps AC, Agius PA, Zhou J, Brunt S, Chachage M, Angelovich TA (2017). Persistence of activated and adaptive-like nk cells in HIV+ individuals despite 2 years of suppressive combination antiretroviral therapy. Front Immunol.

[10] Xu H, Jiang J, Chen W, Li W, Chen Z (2019). Vascular macrophages in atherosclerosis. J Immunol Res.

[11] Nikitskaya E, Lebedeva A, Ivanova O (2016). Cytomegalovirus-productive infection is associated with acute coronary syndrome. J Am Heart Assoc.

[12] Lee S, Doualeh M, Affandi JS, Makwana N, Irish A, Price P (2019). Functional and clinical consequences of changes to natural killer cell phenotypes driven by chronic cytomegalovirus infections. J Med Virol.

[13] Makwana N, Waters S, Irish A, Howson P, Price P (2019). Deciphering effects of uncontrolled cytomegalovirus replication on immune responses in cytomegalovirus DNA-positive renal transplant recipients. Viral Immunol.

[14] Karim B, Wijaya IP, Rahmaniyah R, Ariyanto I, Waters S, Estiasari R (2017). Factors affecting affect cardiovascular health in Indonesian HIV patients beginning ART. AIDS Res Ther.

[15] McKenna SD, Vergilis K, Arulanandam ARN, Weiser WY, Nabioullin R, Tepper MA (2004). Formation of human IFN-beta complex with the soluble type I interferon receptor IFNAR-2 leads to enhanced IFN stability, pharmacokinetics, and antitumor activity in xenografted SCID mice. J Interferon Cytokine Res.

[16] Sottini A, Ghidini C, Serana F, Chiarini M, Valotti M, Badolato R (2008). Decreased type I interferon receptor-soluble isoform in antiretroviral-treated HIV-positive children. J Int Cytokine Res.

[17] Ariyanto IA, Estiasari R, Edwar L, Makwana N, Lee S, Price P (2019). Characterization of natural killer cells in HIV patients beginning therapy with a high burden of cytomegalovirus. Immunol Invest.

[18] Monaco G, Chen H, Poidinger M, Chen J, de Magalhaes JP, Larbi A (2016). flowAI: automatic and interactive anomaly discerning tools for flow cytometry data. Bioinformatics.

[19] Staats J, Divekar A, McCoy JP, Maecker HT (2019). Guidelines for gating flow cytometry data for immunological assays. Methods Mol Biol.

[20] Holder KA, Lajoie J, Grant MD (2018). Natural killer cells adapt to cytomegalovirus along a functionally static phenotypic spectrum in human immunodeficiency virus infection. Front Immunol.

[21] Costa-Garcia M, Ataya M, Moraru M, Vilches C, Lopez-Botet M, Muntasell A (2019). Human cytomegalovirus antigen presentation by HLA-DR+ NKG2C+ adaptive NK cells specifically activate polyfunctional effector memory CD4+ T lymphocytes. Front Immunol.

[22] Ariyanto IA, Estiasari R, Waters S, Wulandari EAT, Fernandez S, Lee S (2018). Active and persistent cytomegalovirus infections affect T cells in young adult HIV patients commencing antiretroviral therapy. Viral Immunol.

