# Supplementary figures and images for: Which NK cell populations mark the high burden of CMV present in all HIV patients beginning ART in Indonesia?
Source: AIDS Res Ther. 2022 Mar 15;19:16. doi: 10.1186/s12981-022-00439-2 (PMC8922863; doi:10.1186/s12981-022-00439-2)

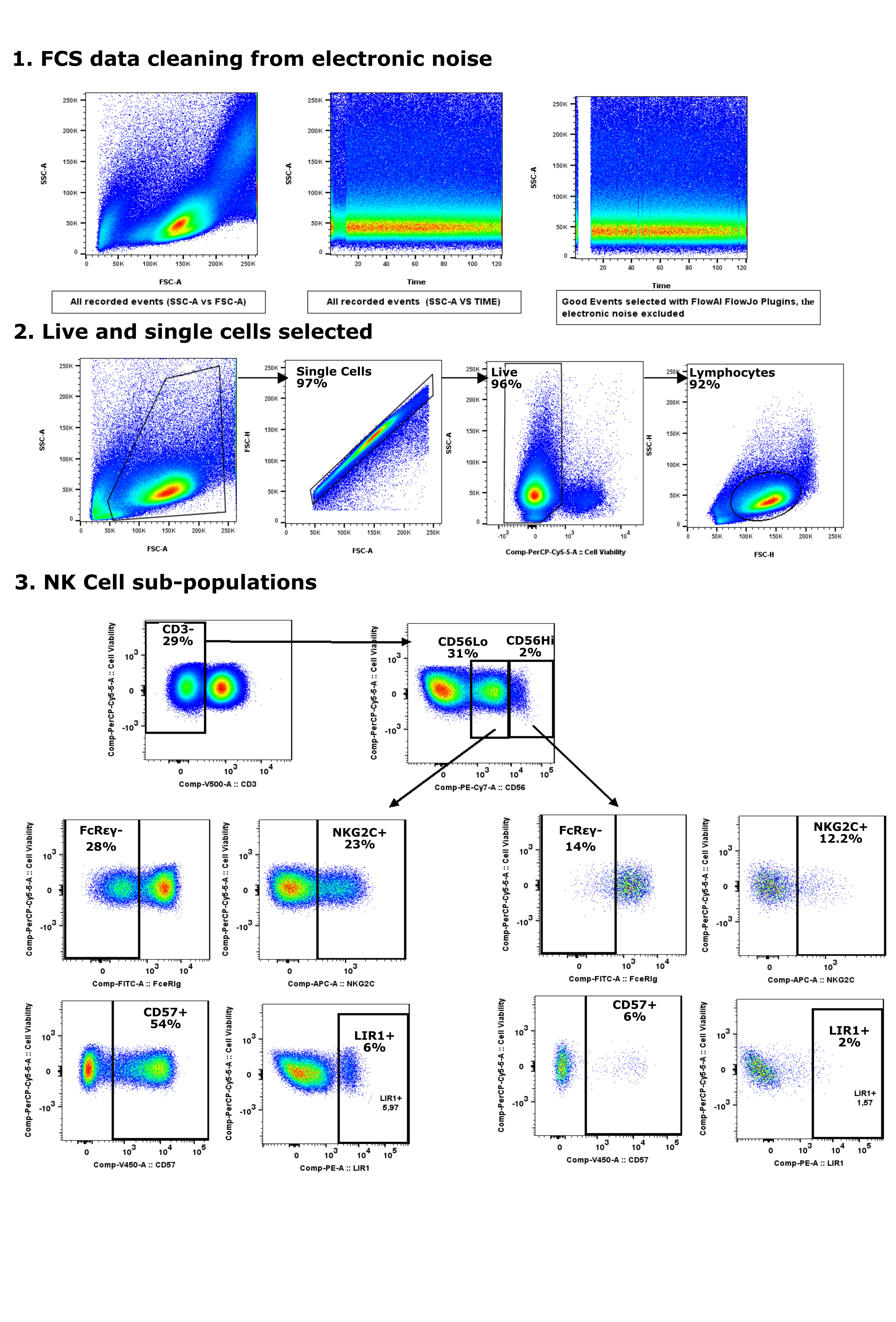

Supplement: Supplementary file 1 — Additional file 1. Gating strategies used to 1) exclude electronic noise; 2) define live and single cells; and 3) define NK cell sub-populations using antibodies defined in Materials and Methods. [file 12981_2022_439_MOESM1_ESM.tif]
